# Exploring Scopoletin's Therapeutic Efficacy in DSS-Induced Ulcerative Colitis: Insights into Inflammatory Pathways, Immune Modulation, and Microbial Dynamics

**DOI:** 10.1007/s10753-024-02048-9

**Published:** 2024-06-26

**Authors:** Abdelrahim Alqudah, Esam Qnais, Omar Gammoh, Yousra Bseiso, Mohammed Wedyan, Mohammad Alqudah, Alaa A. A. Aljabali, Murtaza Tambuwala

**Affiliations:** 1 Department of Clinical Pharmacy and Pharmacy Practice, Faculty of Pharmaceutical Sciences, The Hashemite University, Zarqa, Jordan; 2 Department of Biology and Biotechnology, Faculty of Science, The Hashemite University, Zarqa, Jordan; 3 Department of Clinical Pharmacy and Pharmacy Practice, Faculty of Pharmacy, Yarmouk University, Irbid, Jordan; 4 Physiology Department, School of Medicine and Biomedical Sciences, Arabian Gulf University, Manama, Bahrain; 5 Department of Physiology and Biochemistry, Faculty of Medicine, Jordan University of Science and Technology, Irbid, Jordan; 6 Department of Pharmaceutics and Pharmaceutical Technology, Faculty of Pharmacy, Yarmouk University, Irbid, 21163 Jordan; 7 College of Pharmacy, Ras Al Khaimah Medical and Health Sciences University, Ras Al Khaimah, United Arab Emirates; 8 Lincoln Medical School, University of Lincoln, Brayford Pool Campus, Lincoln, LN6 7TS UK

**Keywords:** ulcerative colitis, scopoletin, DSS-induced Colitis, PPARγ, NLRP3 inflammasome

## Abstract

This study aimed to investigate the therapeutic potential of scopoletin in ulcerative colitis, with a primary focus on its impact on crucial inflammatory pathways and immune responses. A male mouse model of DSS-induced colitis was employed with six distinct groups: a control group, a group subjected to DSS only, three groups treated with varying scopoletin doses, and the final group treated with dexamethasone. The investigation included an assessment of the effects of scopoletin on colitis symptoms, including alterations in body weight, Disease Activity Index (DAI), and histopathological changes in colonic tissue. Furthermore, this study scrutinized the influence of scopoletin on cytokine production, PPARγ and NF-κB expression, NLRP3 inflammasome, and the composition of intestinal bacteria. Scopoletin treatment yielded noteworthy improvements in DSS-induced colitis in mice, as evidenced by reduced weight loss and colonic shortening (p < 0.05, < 0.01, respectively). It effectively diminished TNF-α, IL-1β, and IL-12 cytokine levels (p < 0.01, p < 0.05), attenuated NLRP3 inflammasome activation and the associated cytokine release (p < 0.05, p < 0.01), and modulated the immune response by elevating PPARγ expression while suppressing NF-κB pathway activation (p < 0.05, p < 0.01). Additionally, scopoletin induced alterations in the gut microbiota composition, augmenting beneficial Lactobacillus and Bifidobacteria while reducing E. coli (p < 0.05). It also enhanced tight junction proteins, signifying an improvement in the intestinal barrier integrity (p < 0.05, < 0.01). Scopoletin is a promising therapeutic agent for managing ulcerative colitis, showing benefits that extend beyond mere anti-inflammatory actions to encompass regulatory effects on gut microbiota and restoration of intestinal integrity.

**Supplementary Information:**

The online version contains supplementary material available at 10.1007/s10753-024-02048-9.

## Introduction

Ulcerative colitis (UC), a major subtype of inflammatory bowel disease (IBD), is a chronic and often debilitating condition that primarily affects the colon and rectum. It is characterized by episodes of inflammation and ulceration of the colonic mucosa [1]. UC's clinical manifestations of UC include abdominal pain, diarrhea, and rectal bleeding, which significantly affect the patients' quality of life. The pathogenesis of UC is multifactorial and involves an interplay between genetic predispositions, environmental triggers, and dysregulated immune responses [2].

Central to the pathophysiology of UC is the dysregulation of several key molecular pathways that are crucial for the maintenance of gut homeostasis and development of inflammation. The Peroxisome Proliferator-Activated Receptor Gamma (PPARγ) plays a significant role in intestinal inflammation [3]. As a nuclear receptor, PPARγ regulates genes involved in lipid metabolism, glucose homeostasis, and immune responses [4]. In UC, the anti-inflammatory action of PPARγ becomes critical because its activation can lead to a decrease in the production of inflammatory mediators, thus offering a protective mechanism against excessive inflammation [5].

Conversely, the Nuclear Factor kappa-light-chain-enhancer of activated B cells (NF-κB) pathway is a key regulator of the immune response and has been found to be persistently activated in UC patients [6]. This activation leads to the overproduction of inflammatory cytokines such as Tumor Necrosis Factor-alpha (TNF-α), Interleukin-1 beta (IL-1β), and Interleukin-12 (IL-12), which are crucial in perpetuating the inflammatory process [7]. In particular, TNF-α is a central mediator in UC and contributes to the inflammatory cascade and mucosal damage [8]. The roles of IL-1β and IL-12 are equally pivotal; IL-1β is involved in the amplification of the inflammatory response, whereas IL-12 is instrumental in the differentiation of T cells and the propagation of the Th1 immune response, which is implicated in the pathogenesis of UC [9].

Another important aspect of UC pathophysiology involves the NLR family pyrin domain-containing 3 (NLRP3) inflammasome [10]. Activation of the NLRP3 inflammasome leads to the production of the pro-inflammatory cytokines IL-1β and IL-18, contributing to the chronic inflammatory state seen in UC [11]. Dysregulation of the NLRP3 inflammasome is linked to the ongoing inflammatory response and highlights the complex nature of immune regulation in UC [12]. Thus, understanding these molecular interactions is vital for the identification of novel therapeutic targets.

The integrity of the intestinal epithelial barrier plays a pivotal role in maintaining gut homeostasis and in protecting against pathogenic microorganisms and inflammatory stimuli. Disruptions in this barrier, characterized by alterations in tight junction proteins such as occludin and ZO-1, lead to increased intestinal permeability, a condition often referred to as "leaky gut." This compromised barrier function facilitates the translocation of bacteria and antigens, contributing to the chronic inflammation observed in UC [13]. Concurrently, dysbiosis, defined as an imbalance in gut microbiota composition, is a significant factor in UC pathogenesis. Patients with UC often exhibit a reduction in microbial diversity, including lower levels of beneficial bacteria, such as Lactobacillus and Bifidobacteria, and higher levels of potentially harmful bacteria, such as Escherichia coli. This imbalance can exacerbate mucosal inflammation through mechanisms such as alterations in short-chain fatty acid production, impaired barrier function, and immune system dysregulation [14, 15]. Collectively, these findings underscore the importance of epithelial barrier integrity and microbial balance in the gut, highlighting their role in the complex interplay between genetic, environmental, and microbial factors in UC pathogenesis.

Scopoletin (SCP), or 1, 6-methoxy-7-hydroxy coumarin, is a naturally occurring phytochemical known for its wide range of pharmacological effects, including anticancer [16], antidiabetic [17], anti-inflammatory [18], cardioprotective [19], and hepatoprotective [20] activities. Notably, the antioxidant properties of scopoletin make it a promising candidate for treating ROS-mediated diseases [21]. Previous *in vitro* studies on Raw 264.7 macrophages have shown the ability of scopoletin to inhibit the production of key cytokines, including TNF-α, IL-1β, and IL-6 [22]. Further *in vivo* research utilizing scopoletin isolated from Hypochaeris radicata and Crossosthepium clinensis roots explored its effects on inflammation and acute oral toxicity [21]. This study revealed that scopoletin, administered orally through methanolic root extracts, effectively normalized the cytokine expression disrupted by inflammation. Additionally, the role of scopoletin in modulating CD64 expression and enhancing phagocytosis under inflammatory conditions underscores its potential as an immunomodulatory agent [23].

This study aimed to explore the therapeutic potential of scopoletin in ulcerative colitis, focusing on its effects on the key molecular pathways involved in UC pathophysiology. It seeks to understand the modulatory role of scopoletin on PPARγ and NF-κB and the production of cytokines such as TNF-α, IL-1β, and IL-12, which are critical in the inflammatory process of UC. Additionally, this study investigated the impact of scopoletin on the NLRP3 inflammasome, assessing its potential in reducing the chronic inflammation characteristic of UC. The overarching goals are to delineate the role of scopoletin in UC's molecular pathways of UC, evaluate its effectiveness as a novel therapeutic agent, and contribute to the development of targeted treatments for UC.

## Materials and Methods

### Experimental Animals

In this study, male mice aged 7–8 weeks and weighing–23–26 g were used. These mice were obtained from the animal house facility at the Hashemite University. They were kept under controlled conditions in the animal house, with regular access to food and water and a 12-h light-dark cycle. Before starting the experiments, mice were allowed to adapt to the laboratory environment for at least 2 h. Each mouse was used only once in this study. The study adhered to the Ethics Committee of Hashemite University's approval (IRB number: 14/4/2021/2022, dated April 14, 2022) and followed the ARRIVE guidelines for ethical animal research reporting.

### Colitis Induction and Study Design

To induce acute colitis, mice were administered 2.5% (w/v) DSS (Sigma-Aldrich, Gillingham, Dorset, UK) in drinking water for 7 days. The mice were divided into six groups of ten mice each. The control group received water only. DSS group was given DSS. Scopoletin-treated groups with different doses of (1 mg/kg, 1.5 mg/kg, and 2 mg/kg, respectively) along with DSS.Dexamethasone (2 mg/kg) was orally administered to the final group as a standard reference drug. The doses of scopoletin (1, 1.5, and 2 mg/kg) were chosen based on the preliminary data (Supplementary Fig. 1). A pilot experiment conducted prior to the main study assessed the dises activity index (DAI) across a range of doses of scopoletin in a subacute model of colitis. These preliminary investigations revealed that doses below 1 mg/kg were less effective in ameliorating colitis symptoms, whereas doses exceeding 2 mg/kg did not provide additional therapeutic benefits, and were associated with an increased risk of toxicity. Therefore, the selected doses were deemed to offer an optimal balance between efficacy and safety.

Socopoletin was administered orally to Groups III to V, starting 14 days prior to and during the DSS treatment, once daily. Group VI was administered with socopoletin (2 mg/kg) throughout the experiment. The control and DSS groups were administered with water instead of socopoletin.

The weights of the mice were recorded on specific days (0, 7, 14, 15, 16, 17, 18, 19, 20, and 21) by using an electronic analytical balance (Sartorius Products, Germany). The Disease Activity Index (DAI) was evaluated as described by Alex *et al.* [24]. Briefly, a comprehensive scoring system was employed to quantitatively assess the key clinical parameters of disease activity, including body weight loss, stool consistency, rectal bleeding, and the overall clinical condition of the mice. Each parameter was assigned a score, as follows:

- **Body Weight Loss**: Scores were assigned based on the percentage of weight loss compared to baseline: 0% (score 0), 1–5% (score 1), 5–10% (score 2), 10–15% (score 3), and > 15% (score 4).
- **Stool Consistency**: Normal stools were scored as 0, soft but formed stools as 1, very soft stools as 2, and liquid stools as 3.
- **Rectal Bleeding**: No scored as 0, hemoccult positive as 1, visible blood traces in the stool as 2, and gross bleeding, 3.
- **Overall Clinical Condition**: Mice were observed for signs of distress, including activity level and posture, with scores ranging from 0 (normal behavior) to 3 (severe distress).

The cumulative Disease Activity Index (DAI) was calculated by summing the scores of these parameters, providing a comprehensive assessment of colitis severity ranging from 0 (no disease) to 13 (maximum disease severity).

After 21 days, the mice were euthanized by cervical dislocation under anesthesia. Their colons, from the cecum to 1 cm above the anus, were measured and prepared for histological examination with hematoxylin and eosin (H&E) staining using a previous scoring system.

### Myeloperoxidase (MPO) Assay

MPO assay was utilized to evaluate MPO activity, serving as an indicator of neutrophil presence and distribution in tissue samples. Initially, the colonic tissues were weighed and homogenized in phosphate-buffered saline (PBS) at a predetermined ratio of 1:9, based on the tissue weight to PBS volume.

After homogenization, the mixture was centrifuged to separate the supernatant containing MPO. Supernatants were collected for further analysis. The measurement of MPO activity proceeded according to the prescribed procedures of the MPO assay kit's manufacturer (Abcam, Cambridge, UK). Adhering to these standardized instructions is vital for the consistent and precise evaluation of MPO activity, thereby providing an accurate gauge of neutrophil infiltration levels in the colonic tissues.

### Intestinal Bacteria Cultivation and Quantitation

The objective of the experiment was to quantify the specific intestinal bacteria on the final day (day 21). Fecal pellets were collected, weighed, and homogenized in 1 ml of sterile PBS. This homogenate was then subjected to a sequence of serial dilutions that were carefully plated onto distinct media, each tailored to different bacterial types. These included MRS medium for lactobacilli, BSM Medium for bifidobacteria, and MacConkey Agar for gram-negative bacteria, all sourced from Sigma-Aldrich (Gillingham, Dorset, UK).

Plated cultures were then incubated under meticulously regulated conditions suitable for growth. Cultures on MRS and BSM, aimed at cultivating lactobacilli and bifidobacteria, respectively, were incubated anaerobically for 2–3 days at 37 °C. Conversely, MacConkey Agar plates, designed for gram-negative bacteria, underwent aerobic incubation, but only overnight at 37 °C incubation, colonies displaying the characteristic features of their respective bacterial groups were counted. For a valid and comprehensive count of the bacterial colonies, only plates with colony counts ranging from 30 to 300 were considered. This specific range was chosen to circumvent potential errors, such as overcounting in densely populated plates or undercounting in sparsely populated plates. Thus, this approach ensured a reliable measure of bacterial populations in fecal matter.

### Preparation of Caecal Bacterial Lysates

The Caecal Bacterial Lysates (CBL) were prepared according to the methodology described by Dieleman *et al.* [25]. This commenced with the extraction of cecal content from the mice, which was then thoroughly mixed in RPMI 1640 medium through vigorous vortexing. This step ensured a homogeneous suspension of cecal material in the medium. Subsequently, the mixture was incubated with 10 μg/ml DNase and 0.01 M MgCl2. The inclusion of DNase is crucial for degrading any DNA present, whereas MgCl2 serves as a catalyst for enhancing the enzymatic function of DNase incubation, the sample underwent for 3-min homogenization using 0.1 mm glass beads in a Mini-Bead Beater. This method, which utilizes glass beads, is a standard method for physically breaking bacterial cell walls, thereby releasing cellular content into a homogenate. The homogenate was then centrifuged at 10,000 × g for 10 min, a critical step for separating bacterial cell debris from the supernatant containing bacterial lysates.

The final stage involved refining the supernatant by passing it through a 0.45 μM syringe filter. This filtration process effectively removed any residual particulate matter and yielded a clear lysate. The resulting filtered CBL was set aside for subsequent experimental use or analysis as required by the study.

### Mesenteric Lymph Node Cell Cultures

Mesenteric lymph nodes (MLN) from mice were meticulously harvested following the method outlined by Ruyssers *et al.* [26]. The initial phase of this process involves the creation of single-cell suspensions from the lymph nodes. This step is critical because it ensures that the cells are individually separated, thereby facilitating their independent analysis or utilization in further experiments.

Once the single-cell suspensions were established, approximately 4 × 10^5^^5 MLN cells were extracted and mixed with 20 μg/ml of Caecal Bacterial Lysates (CBL). The mixture was then placed in 96-well flat-bottom microplates. The microplates were filled with RPMI 1640 medium supplemented with 5% heat-inactivated fetal calf serum and 50 mg/ml gentamicin. Fetal calf serum provides vital nutrients and growth factors necessary for cell sustenance, while gentamicin helps prevent bacterial contamination within the culture.

Subsequently, these cultures were incubated in a carefully controlled environment that was optimal for cell culture. This environment was maintained at 37 °C in a 5% CO2 atmosphere, closely simulating natural bodily conditions, to foster cell growth and survival. The incubation duration was set at 72 h, allowing ample time for the cells to interact with CBL and trigger potential cellular responses.

Following the incubation period, the next critical stage involved the collection of culture supernatants. These supernatants are significant because they contain cytokines secreted by MLN cells in reaction to CBL. Cytokines play a vital role as indicators of immune responses, making them a primary focus in experimental settings. The collected supernatants were preserved at -20 °C for later analysis. Storing the samples at this temperature is crucial for maintaining the integrity of cytokines and other components in the supernatants, ensuring their stability for precise and accurate cytokine measurements in subsequent evaluations.

### Cytokine Assays

For cytokine analysis, the colon samples were first weighed and then meticulously blended with phosphate-buffered saline (PBS) at a ratio of 1 part sample to 9 parts PBS while being maintained on ice. This step ensured the proper dilution and preservation of the samples for analysis. Subsequently, the blended mixture was centrifuged at 2, 000 × g for 40 min at a controlled temperature of 4 °C. This centrifugation step was critical for separating the supernatant, which contained the analytes of interest.

The supernatant obtained was carefully extracted and stored at − -20 °C for future analyses. This freezing step is crucial for preserving the integrity of the substances contained within the supernatant.

To specifically measure the concentrations of certain cytokines, namely TNF-α, IL-1β, and IL-12, enzyme-linked immunosorbent assay (ELISA) kits were used. These kits, sourced from Abcam (Cambridge, UK), were designed for precise cytokine quantification. These kits were used in strict accordance with the manufacturer's guidelines and procedures, ensuring the standardization and reliability of the results. By following these detailed protocols, this study aimed to accurately determine the levels of these cytokines, which are key indicators of inflammatory responses in colon tissue samples.

### Quantitative Real-time Polymerase Chain Reaction (qRT-PCR)

In the quantitative real-time polymerase chain reaction (qRT-PCR) phase of the study, total RNA was extracted from mouse colon tissue samples using the TRIzol reagent. To ensure the quality of the extracted RNA, spectrophotometric measurements were taken at 260/280 nm wavelengths. This step is crucial as it assesses both the concentration and purity of the RNA, which are key factors for accurate downstream analysis.

Once the RNA quality was confirmed, it was then converted into complementary DNA (cDNA) using the Revert Aid First Strand cDNA Synthesis Kit (Thermo Scientific, USA). This conversion was essential for the subsequent qRT-PCR process, as cDNA served as the template for amplification.

The qRT-PCR was performed using the 7500 Fast Real-Time PCR System (Applied Biosystems, USA), coupled with the SYBR Green Plus reagent kit (Roche, UK). SYBR Green is a widely used dye in PCR assays because it binds to double-stranded DNA and allows for the detection of amplified gene products in real time. The target mRNA molecules for this procedure are TNF-α, IL-1β, IL-12, ZO-1, occludin, and β-actin. β-actin mRNA was included as an internal standard, serving as a baseline for normalization of the data. Specific primer sequences used for each target gene are listed in Table 1.

**Table 1:** Oligonucleotide primers used for qRT-PCR

Gene	Primer sequence
TNF-α	Sense: 5′- GCCTCCCTCTCATCAGTTCTA-3′
	Anti-sense: 5′- GGCAGCCTTGTCCCTTG-3′
IL-1β	Sense: 5′- ACCTGTGTCTTTCCCGTGG-3′
	Anti-sense: 5′- TCATCTCGGAGCCTGTAGTG-3′
IL-12	Sense: 5′- GGTCACACTGGACCAAAGGGACTATG-3′
	Anti-sense: 5′- ATTCTGCTGCCGTGCTTCCAAC-3′
ZO-1	Sense: 5’-GCGGGGCGGACGCTT-3’
	Anti-sense: 5’-AAATCCAAACCCAGGAGCCC-3’
Occludin	Sense: 5’-TGTCCGTGAGGCCTTTTGAA-3’
	Anti-sense: 5’-AGAGTACGCTGGCTGAGAGA-3’
β-actin	Sense: 5′-CTACCGTCGTGACTTCGC-3′
	Anti-sense: 5′- GGGTGACATCTCCCTGTT-3′

### Western Blotting Analysis

In the Western blotting analysis phase of the study, the process began with the homogenization of colon tissue samples, which is essential for breaking down cell structures and releasing proteins. Total protein was extracted from homogenized samples. A Pierce BCA protein assay kit (Thermo, USA) was used to determine the concentrations of the extracted proteins. After quantifying the protein content, the protein lysates were separated on 12% sodium dodecyl sulfate-polyacrylamide gel electrophoresis gels. The separated proteins were transferred to polyvinylidene difluoride (PVDF) membranes (Bio-Rad, Hercules, CA, UK).

To prevent nonspecific binding of antibodies to the membrane, blocking was performed using a buffer containing 5% non-fat milk for 2 h. Following blocking, the membranes were incubated overnight at 4 °C with primary antibodies targeting the specific proteins of interest: PPARγ, p-P65, p-IkB, NLRP3, ASC, IL-1β, and caspase-1 (Abcam, UK).

After primary antibody incubation, membranes were incubated with the corresponding secondary antibodies for 1 h at room temperature. Blots were detected using a western blotting detection system (ChemiDoc, Bio-Rad, Hercules, CA, UK) and the intensity of the bands was quantified using image J software. For accurate normalization of the results, β-actin (Abcam, Cambridge, UK) was used as an internal control.

### Assessment of Mucosal Barrier of Colonic Epithelial Cells

In this study, quantification of occludin and ZO-1 levels in colonic tissues was performed using qPCR and commercially available ELISA kits (Mybiosource, CA, USA). These kits were specifically designed to accurately measure these proteins. Adhering to the manufacturer's guidelines is crucial to ensure the precision and reliability of the results. Occludin and ZO-1 are important, as they are key markers of intestinal barrier integrity, and their quantification provides valuable insights into the condition of colonic tissues in the context of this study.

### Statistical Analysis

The data were statistically analyzed using GraphPad Prism software. To evaluate differences in mean values of normally distributed data, one-way ANOVA was utilized, complemented by Dunnett’s test for post hoc analysis. Statistical significance was determined at a p-value threshold of ≤ 0.05, indicating a significant difference between the compared groups or conditions.

## Results

### Attenuation of DSS-Induced Mouse Experimental Colitis by Scopoletin

**Fig. 1:**
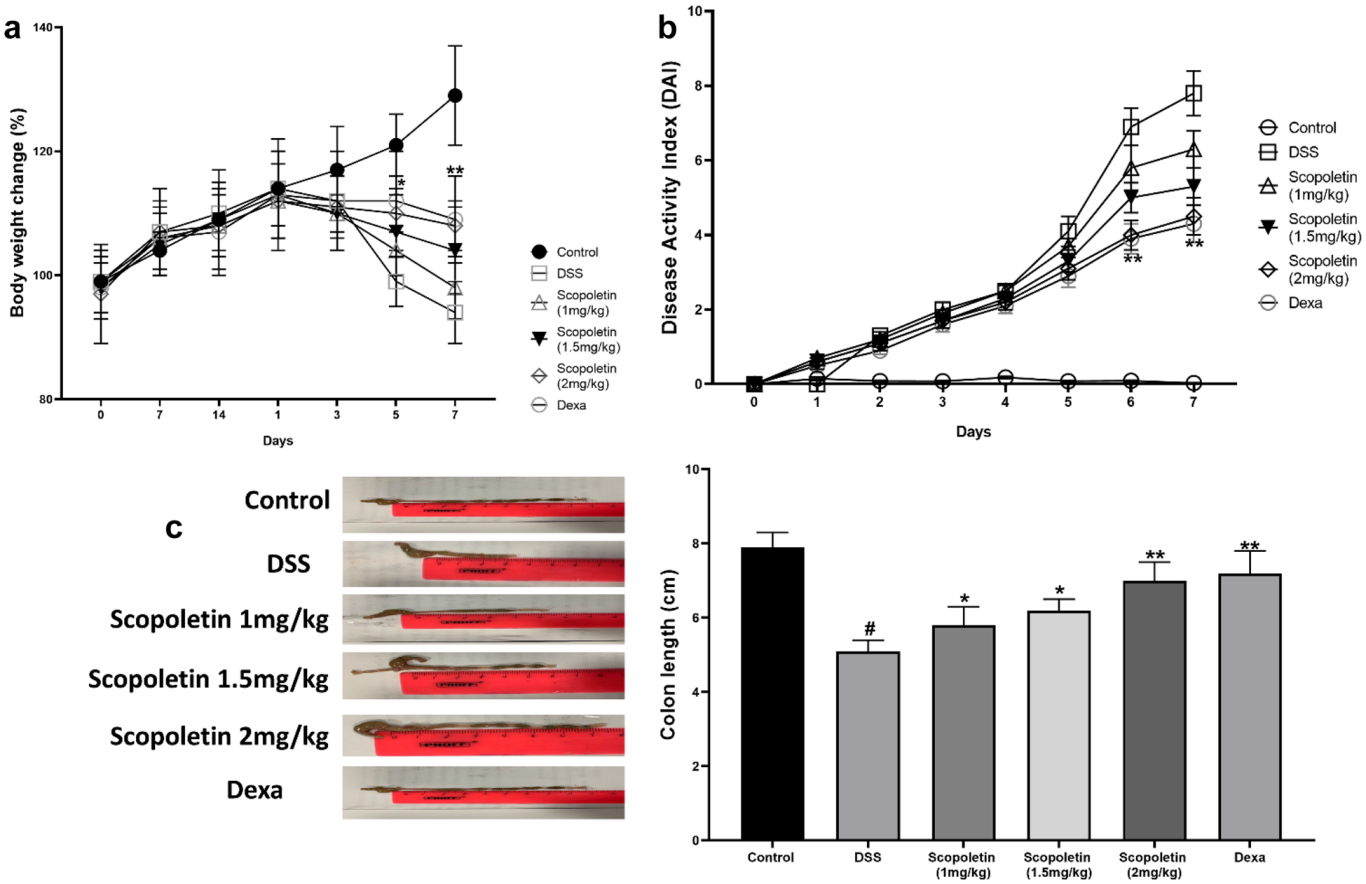
Scopoletin treatment led to a reduction in the vulnerability of mice towards DSS-induced experimental colitis. **a** displays the change in body weight for each group, while **b** shows the Disease Activity Index (DAI). Additionally, **c** illustrates the measured lengths of colons from each group of mice. The data are presented as means ± SEM (n = 10 per group). Significant differences were denoted as ∗p < 0.05 and ∗∗p < 0.01 compared to the DSS-treated group, while #p < 0.05 indicates significance compared to the control group. Dexa; dexamethasone.

**Fig. 2:**
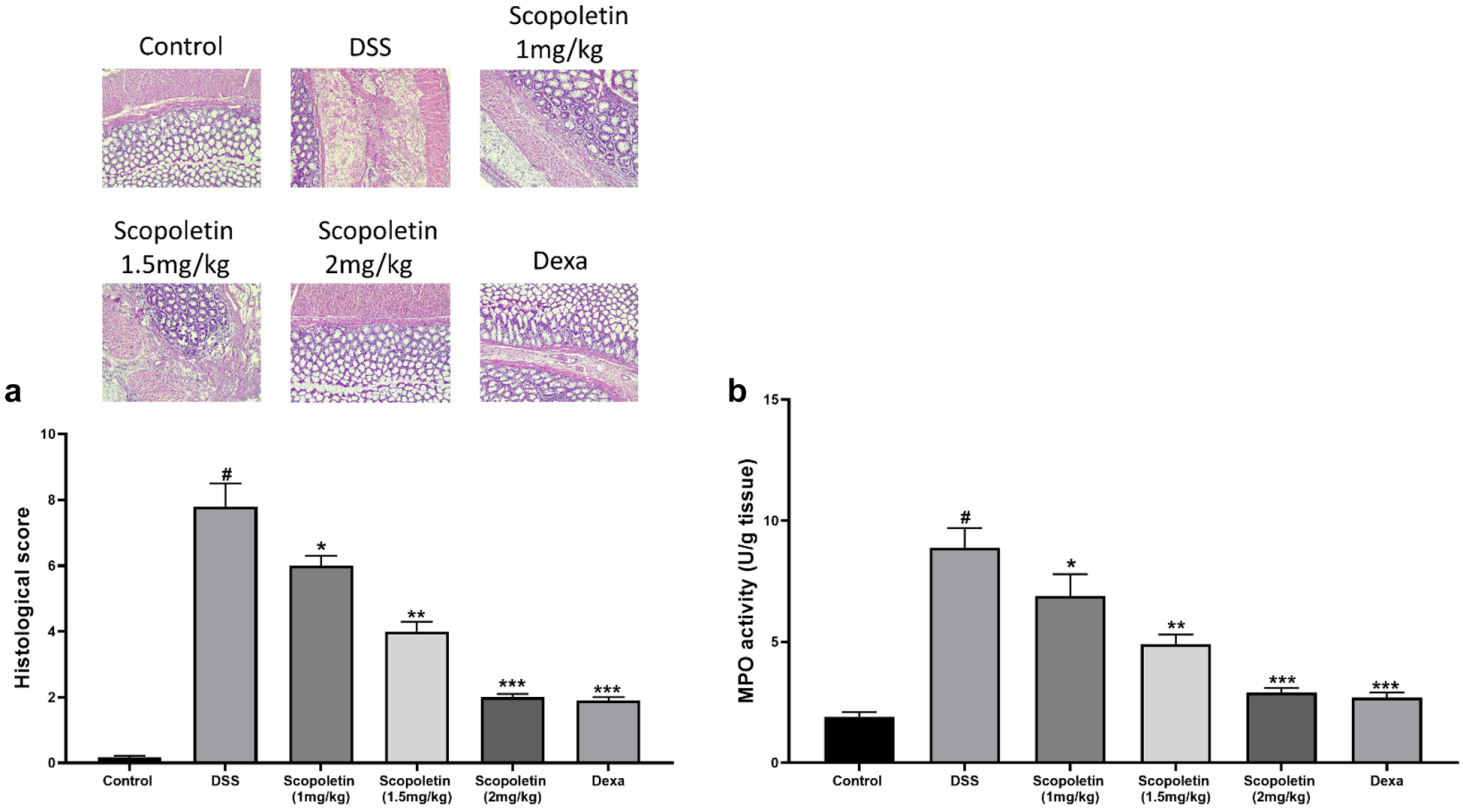
Scopoletin treatment effectively protected mice against colon damage induced by DSS. **a** colons from each experimental group underwent histological evaluation using H&E staining at ×200 magnification, alongside histopathological scoring. **b** indicates the detected MPO (myeloperoxidase) activity in the colonic tissues. The data are presented as means ± SEM (n = 10). Statistical significance was marked as ∗p < 0.05,  ∗∗p < 0.01, and ***<0.001 compared to the group treated with DSS alone, while #p < 0.05 indicates significance compared to the control group. Dexa; dexamethasone.

Our study revealed that DSS-induced colitis in mice typically results in significant body weight loss, diarrhea, and bloody stools. Notably, the administration of scopoletin at doses of 1, 1.5, and 2 mg/kg and dexamethasone markedly alleviated weight loss during colitis progression at weeks 5 and 7 (Fig. 1a, n = 10, p < 0.05, < 0.01, respectively). The Disease Activity Index (DAI), which reflects the severity of weight loss, stool consistency, and blood presence in stools, showed a substantial decrease in the scopoletin-treated groups and dexamethasone –treated group compared to the DSS-only group at weeks 5, 6, and 7 (Fig. 1b, n = 10, p < 0.05, < 0.01, respectively). Furthermore, scopoletin and dexamethasone treatment significantly counteracted the colonic shortening typically caused by DSS-induced colitis (Fig. 1c, n = 10, p < 0.05, < 0.01, respectively). Histopathological examination also indicated less crypt distortion, goblet cell loss, mononuclear cell infiltration, and mucosal damage in the colons of scopoletin-treated and dexamethasone-treated mice (Fig. 2a, n = 10, p < 0.05, < 0.01, respectively). Scopoletin and dexamethasone also notably diminished the DSS-induced hyperactivation of myeloperoxidase (MPO) (Fig. 2b, n = 10, p < 0.05, < 0.01, respectively). These results suggested that scopoletin effectively reduced the severity of DSS-induced experimental colitis in mice.

### Suppression of Pro-inflammatory Cytokines by Scopoletin

**Fig. 3:**
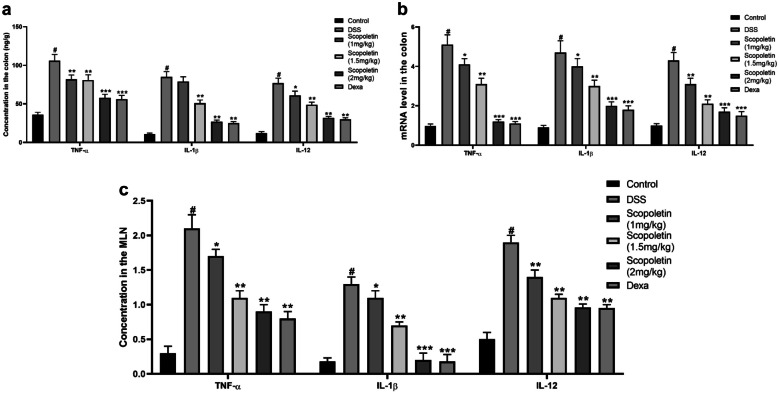
Scopoletin treatment leads to a reduction in pro-inflammatory cytokines in both colonic tissue and Mesenteric Lymph Nodes (MLN). **a** displays the protein levels of TNF-α, IL-1β, and IL-12, while **b** exhibits mRNA levels in colonic tissue. Additionally, **c** shows the levels of TNF-α, IL-1β, and IL-12 in MLN measured through ELISA. The data are presented as means ± SEM. Significance levels were indicated as ∗p < 0.05 and ∗∗p < 0.01 compared to the group treated with DSS alone, while #p < 0.05 denotes significance compared to the control group. Dexa; dexamethasone.

Examination of cytokine expression in the acute DSS colitis model showed a significant increase in TNF-α, IL-1β, and IL-12 levels following DSS exposure (Fig. 3a, n = 10, p < 0.05). Treatment with Scopoletin (1, 1.5, and 2 mg/kg) and dexamethasone significantly reduced the levels of these cytokines (Fig. 3a, n = 10, p < 0.01, < 0.05). Furthermore, the mRNA levels of these cytokines in colonic tissues were also notably lowered by scopoletin treatment, especially at 1.5 and 2 mg/kg doses, and dexamethasone treatment (Fig. 3b, n = 10, p < 0.01). Additionally, Scopoletin and dexamethasone significantly suppressed the release of TNF-α, IL-1β, and IL-12 from mesenteric lymph node (MLN) cells in response to CBL challenge (Fig. 3c, n = 10, p < 0.05, 0.01). Collectively, these findings suggest that scopoletin markedly inhibits elevated levels of these inflammatory cytokines.

### Modulation of PPARγ and NF-κB Expression by Scopoletin

**Fig. 4:**
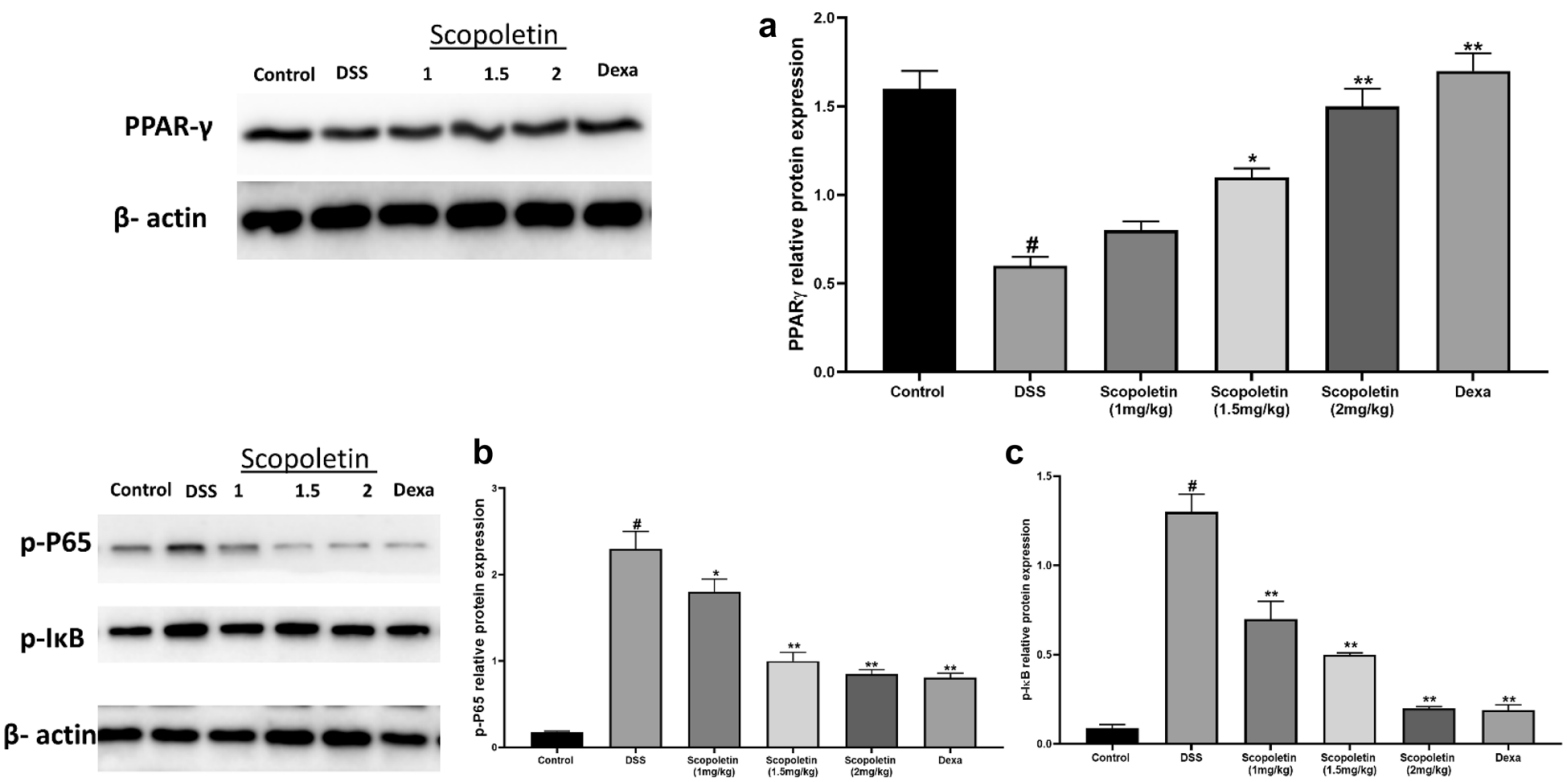
Scopoletin activates PPARγ and downregulates NF-κB in colonic tissue. **a** the level of PPARγ in the colonic tissue, while **b** showcases the analysis of NF-κB p65 and IκB protein levels in the colonic tissue using western blot. β-actin was employed as a control. The data are expressed as means ± SEM. Significance levels were denoted as ∗p < 0.05 and ∗∗p < 0.01 in comparison to the group treated solely with DSS, while #p < 0.05 indicates significance compared to the control group. Dexa; dexamethasone.

We observed that DSS reduced PPARγ expression in the colon, but scopoletin administration (particularly at 1.5 and 2 mg/kg) and dexamethasone administration increased its expression (Fig. 4a, n = 10, p < 0.05, < 0.01). Scopoletin and dexamethasone also reduced the phosphorylation of NF-κB p65 and IκB (Fig. 4b and c, n = 10, p < 0.01). This indicated that scopoletin has potential anti-inflammatory properties through the modulation of NF-κB and PPARγ expression.

### Reduction of NLRP3 Inflammasome Activation by Scopoletin

**Fig. 5:**
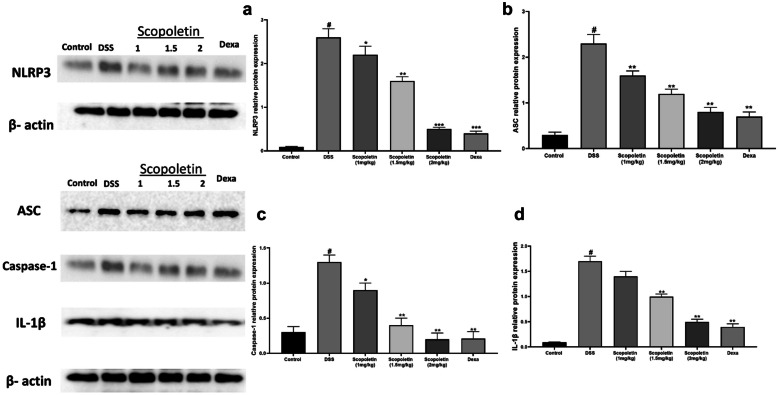
NLRP3 inflammasome activation is decreased in colonic tissue due to scopoletin treatment. The protein levels of NLRP3 (**a**), ASC (**b**), caspase-1 (**c**), and IL-1β (**d**) were assessed via Western blot, utilizing β-actin as a control. The data are represented as means ± SEM. Significant differences were indicated as ∗p < 0.05 and ∗∗p < 0.01 in comparison to the group treated solely with DSS, while #p < 0.05 denotes significance compared to the control group. Dexa; dexamethasone.

Scopoletin treatment significantly reduced the expression of NLRP3 inflammasome components (NLRP3, ASC, and Caspase-1) in a dose-dependent manner (Fig. 5a, b & c, n = 10, p < 0.05, < 0.01), leading to decreased IL-1β release (Fig. 5d, n = 10, p < 0.05, < 0.01). Scopoltein effect was comparable to the reference drug dexamethasone which suggests Scopoletin's efficacy in inhibiting NLRP3 inflammasome activation in DSS-induced colitis.

### Regulation of Intestinal Bacteria by Scopoletin

**Fig. 6:**
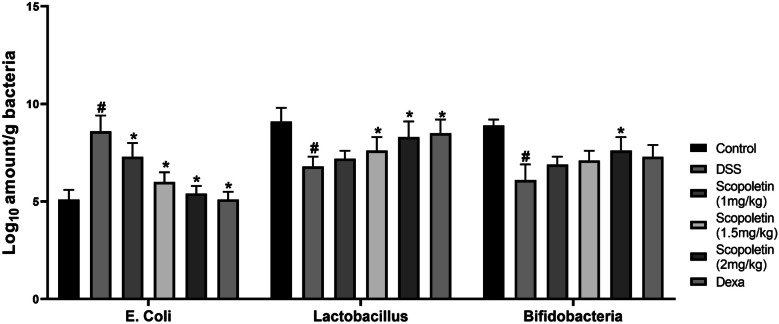
Scopoletin regulates intestinal bacteria in mice. The analysis employed variance analysis using the One-Way ANOVA test. Significance levels were marked as ∗p < 0.05 compared to the corresponding DSS treatment group. Moreover, #p < 0.05 indicate significance compared to the control group within the same treatment group. Dexa; dexamethasone.

Treatment with scopoletin (particularly at 1.5 and 2 mg/kg) and dexamethasone resulted in a significant decrease in commensal E. coli and an increase in beneficial Lactobacillus and Bifidobacteria levels compared to those in the DSS group (Fig. 6, n = 10, p < 0.05). This indicated that scopoletin has a regulatory effect on intestinal bacteria in mice with DSS-induced colitis.

### Protection of Mucosal Barrier by Scopoletin

**Fig. 7:**
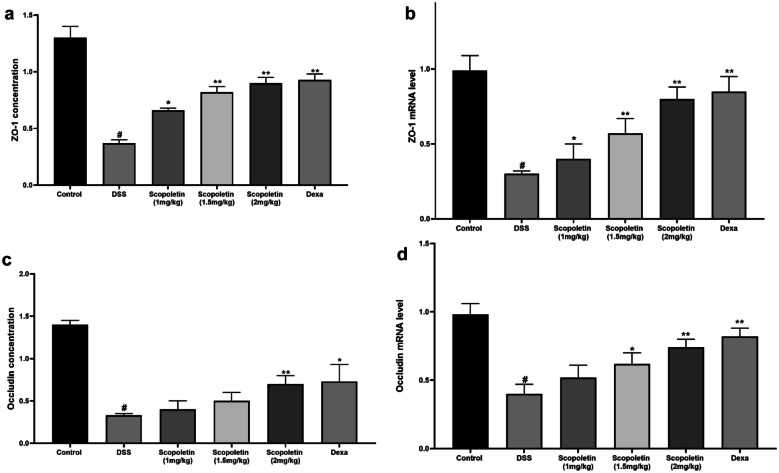
Scopoletin treatment restored tight junction protein in colonic tissue. Scopoletin treatment upregulates ZO-1 concentration and mRNA levels (**a**, **b**) and occludin concentration and mRNA level (**b**, **c**) in colonic tissue. The analysis employed variance analysis using the One-Way ANOVA test. Significance levels were marked as ∗p < 0.05 and ∗∗p < 0.01 compared to the corresponding DSS treatment group. Moreover, #p < 0.05 indicate significance compared to the control group within the same treatment group. Dexa; dexamethasone.

A noteworthy enhancement in tight junction proteins, specifically ZO-1 and occludin, was observed in the scopoletin-treated groups (at 1.5 and 2 mg/kg), suggesting a protective effect on the mucosal barrier of colonic epithelial cells (Fig. 7a–d, n = 10, p < 0.05, < 0.01).

## Discussion

Ulcerative colitis (UC), a chronic and recurrent inflammation of the gastrointestinal tract, is common in developed countries [27]. The primary treatments for UC are anti-inflammatory or immunosuppressive drugs, which are often ineffective and can lead to significant adverse effects. Recently, there has been growing interest in utilizing natural compounds as a means to prevent and treat various illnesses. However, the health advantages and molecular processes associated with these compounds have not been fully explored. Thus, more research is needed to fully understand the health benefits and mechanisms underlying their effects.

In our experiment, mice treated with DSS displayed symptoms similar to those of UC, including weight loss, changes in the Disease Activity Index (DAI), and histopathological signs such as damage to intestinal epithelial cells and the release of inflammatory cytokines. However, socopoletin showed promising results by reducing the symptoms and signs of DSS-induced colonic inflammation. Specifically, socopoletin was effective in reducing DSS-induced pathological damage in the colon caused by DSS. In addition, socopoletin inhibited MPO activity, suggesting its potential to decrease inflammation related to this condition. A notable feature of DSS-induced colitis is the excessive production of pro-inflammatory cytokines, with TNF-α, IL-1β, and IL-12 being key cytokines involved in the mucosal immune response in inflammatory bowel disease (IBD) [28, 29]. Our *in vivo* studies have shown that socopoletin efficiently reduces the secretion of these cytokines in the colonic tissue and MLN in mice with DSS-induced colitis. NF-κB is a crucial signaling molecule in the development of inflammatory diseases and is known to initiate the production of pro-inflammatory cytokines upon activation. Our research evaluated the impact of socopoletin on the NF-κB pathway, revealing that socopoletin significantly inhibited NF-κB activation caused by DSS. This is in agreement with several studies that have demonstrated the anti-inflammatory effects of scopoletin. Initial research indicated that scopoletin could suppress the synthesis of cytokines such as tumor necrosis factor-alpha (TNF-α), interleukin-1β (IL-1β), and interleukin-6 (IL-6) in Raw 264.7 macrophages during *in vitro* experiments [22]. Moreover, when scopoletin, extracted from the methanolic root extract of H. radicata and Crossosthepium clinensis, was administered orally, it reversed the stimulation of cytokine expression caused by inflammation in paw edema, including TNF-α, IL-1β, IL-6, inducible nitric oxide synthase (iNOS), and cyclooxygenase-2 (COX-2) [30].

Furthermore, a study conducted by Desreumaux *et al.* indicated that PPARγ activators can alleviate colon inflammation [31]. PPARγ plays a vital role as an anti-inflammatory mediator in colonic inflammation and there is an established negative interaction between NF-κB and PPARγ, where PPARγ is an upstream inhibitor of NF-κB [32]. Notably, disrupting PPARγ expression in macrophages increases susceptibility to DSS-induced colitis [33]. In this context, our study explored the protective mechanism of socopoletin in UC and found a significant increase in PPARγ expression in socopoletin-treated groups. The results of our study strongly indicate that the protective effect of socopoletin in mice with DSS-induced colonic injury is likely mediated through the activation of PPARγ. This activation has the potential to diminish the activation of the NF-κB pathway, which is typically associated with IBD, and thereby suppresses the expression of pro-inflammatory cytokines such as TNF-α, IL-1β, and IL-12. IL-1β plays a pivotal role in driving effective immune responses. Unlike other acute-phase cytokines, the production of active IL-1β depends on inflammasome activation [34]. NLRP3 inflammasome, a key molecular structure triggered by various pathogenic or damage-related patterns, is central to this process [12]. Once activated, NLRP3 complexes with ASC lead to the activation of pro-caspase-1 [10]. This step is crucial for maturation and release of IL-1β. Research has shown that the NLRP3/ASC/caspase-1 pathway is vital in DSS-induced experimental colitis [35]. In our study, socopoletin significantly reduced the production of IL-1β in the colon induced by DSS, and markedly inhibited the increase in the protein levels of caspase-1, ASC, and NLRP3. These findings suggest that socopoletin combats DSS-induced colitis by dampening NLRP3 inflammasome activation. Our *in vivo* findings implied that the beneficial effect of socopoletin on DSS-induced colitis could be attributed to its ability to inhibit NLRP3 inflammasome activation.

Moreover, the role of gut microbiota in maintaining intestinal permeability is well-established, and an imbalance between beneficial and harmful bacteria is known to contribute to the development of IBD [36]. Previous studies have identified a decrease in beneficial bacteria, such as Lactobacillus and Bifidobacteria, and an increase in E. coli in both human IBD patients and murine models of DSS-induced experimental colitis [14, 15]. In the present study, we analyzed the effect of socopoletin on the composition of intestinal bacteria. We found that socopoletin not only regulated the changes in bacterial composition caused by DSS treatment, reducing the levels of Lactobacillus and Bifidobacteria, but also significantly decreased the DSS-induced elevation in E. coli levels. Integrity of the intestinal barrier is essential for maintaining gut health [13]. A compromised intestinal barrier leads to increased permeability, allowing harmful bacteria to infiltrate the intestinal mucosa and trigger inflammation [37]. Increased intestinal permeability is a well-known issue in patients with IBD [38]. Tight junction proteins (TJ) are critical for regulating intestinal epithelial permeability. Previous studies have identified structural abnormalities in TJ proteins, including a reduction in ZO-1 in patients with IBD, which contributes to altered intestinal permeability [39]. Therefore, targeting TJ proteins is a promising strategy for developing new therapeutic approaches against IBD. In our investigation, the treatment with socopoletin showed promising results in restoring the expression of tight TJ proteins, specifically occludin and ZO-1, in mice with DSS-induced colitis. Our data strongly suggest that socopoletin plays a protective role in maintaining intestinal barrier integrity by sustaining the levels of these crucial TJ proteins, thereby reducing the severity of colitis.

In conclusion, our research suggests that socopoletin treatment, when applied as dietary therapy, can mitigate the detrimental effects of DSS-induced colitis. The protective benefits of socopoletin are not limited to its anti-inflammatory actions; it also has regulatory effects on the gut microbiota in mice. Socopoletin has shown the capability restores gut integrity, highlighting its potential as a viable alternative therapeutic option for the management of ulcerative colitis (UC).

## Conclusion

In summary, this study sheds light on the potential of scopoletin as a therapeutic agent to mitigate the effects of DSS-induced colitis, a chronic and recurrent inflammatory condition prevalent in developed countries. Traditional treatments for ulcerative colitis often exhibit limitations in efficacy and are associated with significant adverse effects, prompting a growing interest in exploring natural compounds for preventive and therapeutic purposes. Our investigation focused on elucidating the health benefits and molecular mechanisms underlying the effects of scopoletin, thereby recognizing the need for further comprehensive research in this domain.

An experiment using a DSS-induced colitis mouse model revealed that mice treated with DSS exhibited symptoms akin to human ulcerative colitis, including weight loss, changes in the Disease Activity Index (DAI), and histopathological signs. Scopoletin demonstrated promising results by effectively reduced these symptoms and ameliorated DSS-induced colonic inflammation. Specifically, scopoletin showed potential in diminishing pathological damage to the colon and inhibiting myeloperoxidase (MPO) activity, suggesting its role in reducing inflammation associated with colitis.

In the context of DSS-induced colitis, pro-inflammatory cytokines, particularly TNF-α, IL-1β, and IL-12, play pivotal roles. Scopoletin treatment significantly reduced the secretion of these cytokines in colonic tissue and mesenteric lymph nodes, indicating its anti-inflammatory effects. Furthermore, scopoletin exhibited inhibitory effects on the NF-κB pathway, a crucial signaling molecule in inflammatory diseases, which is consistent with previous studies highlighting the anti-inflammatory properties of scopoletin.

Our study revealed a substantial increase in PPARγ expression after exploring the protective mechanisms of scopoletin in UC. PPARγ activation is known to suppress the NF-κB pathway, suggesting a potential avenue through which scopoletin exerts its protective effects. Additionally, scopoletin demonstrated the ability dampened the activation of the NLRP3 inflammasome, a key molecular structure involved in IL-1β production, further supporting its anti-inflammatory role in DSS-induced colitis.

This study also investigated the impact of scopoletin on the gut microbiota, recognizing its role in maintaining intestinal permeability. Scopoletin demonstrated regulatory effects on bacterial composition, restoring balance by decreasing harmful E. coli levels and increasing beneficial Lactobacillus and Bifidobacteria. Furthermore, scopoletin exhibited a promising ability to restore the expression of tight junction proteins such as occludin and ZO-1, which are essential for maintaining intestinal barrier integrity.

In conclusion, this study highlighted the potential of scopoletin as a dietary therapeutic option for managing ulcerative colitis. Beyond its anti-inflammatory actions, scopoletin's regulatory effects on the gut microbiota and restoration of gut integrity are promising alternatives in the therapeutic landscape for ulcerative colitis. Future studies should delve deeper into the molecular pathways and clinical applications of scopoletin to validate its potential as a viable therapeutic intervention in this challenging inflammatory condition.

## Supplementary Information

Below is the link to the electronic supplementary material.

Supplementary file1 (DOCX 209 KB)

## Data Availability

Data created/analyzed associated with this research is available from the corresponding author upon reasonable request.

## References

[1] Cosnes, J., C. Gowerrousseau, P. Seksik, and A. Cortot. 2011. Epidemiology and natural history of inflammatory bowel diseases. *Gastroenterology* 140: 1785-1794.e4. 21530745 10.1053/j.gastro.2011.01.055

[2] Kim, D.H., and J.H. Cheon. 2017. Pathogenesis of inflammatory bowel disease and recent advances in biologic therapies. *Immune Network* 17: 25–40. https://synapse.koreamed.org/articles/1033559. Cited 21 Dec 2023. 10.4110/in.2017.17.1.25 PMC5334120 28261018

[3] Dubuquoy, L., C. Rousseaux, X. Thuru, L. Peyrin-Biroulet, O. Romano, P. Chavatte, et al. 2006. PPARγ as a new therapeutic target in inflammatory bowel diseases. *Gut* 55: 1341–9. https://gut.bmj.com/content/55/9/1341. Cited 21 Dec 2023. 10.1136/gut.2006.093484 PMC1860011 16905700

[4] Celinski, K., T. Dworzanski, A. Korolczuk, R. Piasecki, M. Slomka, A. Madro, et al. 2011. Effects of peroxisome proliferator-actvated receptors-γamma ligands on dextran sodium sulphate-induced colitis in rats. *Journal of Physiology and Pharmacology* 62: 347–356. 21893696

[5] Su, C.G., X. Wen, S.T. Bailey, W. Jiang, S.M. Rangwala, S.A. Keilbaugh, et al. 1999. A novel therapy for colitis utilizing PPAR-γ ligands to inhibit the epithelial inflammatory response. *The Journal of Clinical Investigation* 104: 383–389. 10449430 10.1172/JCI7145 PMC408529

[6] Yadav, P.N., Z. Liu, and M.M. Rafi. 2003. A diarylheptanoid from lesser galangal (Alpinia officinarum) inhibits proinflammatory mediators via inhibition of mitogen-activated protein kinase, p44/42, and transcription factor nuclear factor-κB. *Journal of Pharmacology and Experimental Therapeutics [Internet]* 305: 925–31. https://jpet.aspetjournals.org/content/305/3/925. Cited 21 Dec 2023. 10.1124/jpet.103.049171 12626645

[7] Shen, H.M., and V. Tergaonkar. 2009. NFkappaB signaling in carcinogenesis and as a potential molecular target for cancer therapy. *Apoptosis [Internet]* 14: 348–63. https://pubmed.ncbi.nlm.nih.gov/19212815/. Cited 9 Oct 2023. 10.1007/s10495-009-0315-0 19212815

[8] Jang, J., S.M. Kim, S.M. Yee, E.M. Kim, E.H. Lee, H.R. Choi, et al. 2019. Daucosterol suppresses dextran sulfate sodium (DSS)-induced colitis in mice. *International Immunopharmacology* 72: 124–130. 30978647 10.1016/j.intimp.2019.03.062

[9] Wang, J., Y.T. Liu, L. Xiao, L. Zhu, Q. Wang, and T. Yan. 2014. Anti-inflammatory effects of apigenin in lipopolysaccharide-induced inflammatory in acute lung injury by suppressing COX-2 and NF-kB pathway. *Inflammation* 37: 2085–90. 10.1007/s10753-014-9942-x. Cited 7 Aug 2023. 24958013 10.1007/s10753-014-9942-x

[10] Lu, A., V.G. Magupalli, J. Ruan, Q. Yin, M.K. Atianand, M.R. Vos, et al. 2014. Unified polymerization mechanism for the assembly of asc-dependent inflammasomes. *Cell [Internet]* 156: 1193–206. http://www.cell.com/article/S0092867414002001/fulltext. Cited 23 Dec 2023. 10.1016/j.cell.2014.02.008 PMC4000066 24630722

[11] Ammar, R.A., A.F. Mohamed, M.M. Kamal, M.M. Safar, and N.F. Abdelkader. 2022. Neuroprotective effect of liraglutide in an experimental mouse model of multiple sclerosis: role of AMPK/SIRT1 signaling and NLRP3 inflammasome. *Inflammopharmacology [Internet]* 30: 919–34. https://pubmed.ncbi.nlm.nih.gov/35364735/. Cited 27 Sep 2022. 10.1007/s10787-022-00956-6 PMC9135867 35364735

[12] Franchi, L., T. Eigenbrod, R. Muñoz-Planillo, and G. Nuñez. 2009. The inflammasome: a caspase-1-activation platform that regulates immune responses and disease pathogenesis. *Nature Immunology* 10: 241–7. https://www.nature.com/articles/ni.1703. Cited 23 Dec 2023. 10.1038/ni.1703 PMC2820724 19221555

[13] Oshitani, N., K. Watanabe, S. Nakamura, Y. Fujiwara, K. Higuchi, and T. Arakawa. 2005. Dislocation of tight junction proteins without F-actin disruption in inactive Crohn’s disease. *International Journal of Molecular Medicine [Internet]* 15: 407–10. 10.3892/ijmm.15.3.407/abstract. Cited 23 Dec 2023. 15702229

[14] Matsuoka, K., and T. Kanai. 2015. The gut microbiota and inflammatory bowel disease. *Seminars in Immunopathology [Internet]* 37: 47–55. 10.1007/s00281-014-0454-4. Cited 23 Dec 2023. 25420450 10.1007/s00281-014-0454-4 PMC4281375

[15] Håkansson, Tormo-Badia N., A. Baridi, J. Xu, G. Molin, M.L. Hagslätt, et al. 2015. Immunological alteration and changes of gut microbiota after dextran sulfate sodium (DSS) administration in mice. *Clinical and Experimental Medicine [Internet]* 15: 107–20. 10.1007/s10238-013-0270-5. Cited 223 Dec 2023. 24414342 10.1007/s10238-013-0270-5 PMC4308640

[16] Seo, E.J., M. Saeed, B.Y.K. Law, A.G. Wu, O. Kadioglu, H.J. Greten, et al. 2016. *Pharmacogenomics of Scopoletin in Tumor Cells [Internet]* 21: 496. https://www.mdpi.com/1420-3049/21/4/496/htm. Cited 9 Jan 2024. 10.3390/molecules21040496 PMC6273985 27092478

[17] Jang, J.H., J.E. Park, and J.S. Han. 2018. Scopoletin inhibits α-glucosidase *in vitr*o and alleviates postprandial hyperglycemia in mice with diabetes. *European Journal of Pharmacology* 834: 152–156. 30031794 10.1016/j.ejphar.2018.07.032

[18] Ding, Z., Y. Dai, H. Hao, R. Pan, X. Yao, and Z. Wang. 2008. Anti-inflammatory effects of scopoletin and underlying mechanisms. *Pharmaceutical Biology [Internet]* 46: 854–60. 10.1080/13880200802367155. Cited 9 Jan 2024.

[19] Rong, N., R. Yang, I.A.A. Ibrahim, and W. Zhang. 2023. Cardioprotective role of scopoletin on isoproterenol-induced myocardial infarction in rats. *Applied Biochemistry and Biotechnology [Internet]* 195: 919–32. 10.1007/s12010-022-04123-z. Cited 9 Jan 2024. 36227500 10.1007/s12010-022-04123-z

[20] Sharma, S., A. Anand, A. Bhatia, V. Sharma, A.K. Singh, D. Banerjee, et al. 2022. Pharmacological evaluation of scopoletin in the carbon tetrachloride-induced hepatotoxicity model in wistar rats. *Journal of Pharmacy and Bioallied Sciences [Internet]* 14: 201. 10.4103/jpbs.jpbs_333_22. Cited 9 Jan 2024. 37051421 10.4103/jpbs.jpbs_333_22 PMC10084995

[21] Jamuna, S., K. Karthika, S. Paulsamy, K. Thenmozhi, S. Kathiravan, and R. Venkatesh. 2015. Confertin and scopoletin from leaf and root extracts of Hypochaeris radicata have anti-inflammatory and antioxidant activities. *Industrial Crops and Products* 70: 221–230.

[22] Kim, H.J., Jang S. Il, Y.J. Kim, H.T. Chung, Y.G. Yun, T.H. Kang, et al. 2004. Scopoletin suppresses pro-inflammatory cytokines and PGE2 from LPS-stimulated cell line, RAW 264.7 cells. *Fitoterapia* 75: 261–6. 15158982 10.1016/j.fitote.2003.12.021

[23] Alkorashy, A.I., A.S. Doghish, A.I. Abulsoud, M.G. Ewees, T.M. Abdelghany, M.M. Elshafey, et al. 2020. Effect of scopoletin on phagocytic activity of U937-derived human macrophages: Insights from transcriptomic analysis. *Genomics* 112: 3518–3524. 32243896 10.1016/j.ygeno.2020.03.022

[24] Alex, P., N.C. Zachos, T. Nguyen, L. Gonzales, T.E. Chen, L.S. Conklin, et al. 2009. Distinct cytokine patterns identified from multiplex profiles of murine DSS and TNBS-induced colitis. *Inflammatory Bowel Disease [Internet]* 15: 341–52. 10.1002/ibd.20753. Cited 21 Dec 2023. 10.1002/ibd.20753 PMC2643312 18942757

[25] Dieleman, L.A., F. Hoentjen, B.F. Qian, D. Sprengers, E. Tjwa, M.F. Torres, et al. 2004. Reduced ratio of protective versus proinflammatory cytokine responses to commensal bacteria in HLA-B27 transgenic rats. *Clinical and Experimental Immunology [Internet]* 136: 30–9. 10.1111/j.1365-2249.2004.02410.x. Cited 21 Dec 2023. 15030511 10.1111/j.1365-2249.2004.02410.x PMC1808999

[26] Ruyssers, N.E., B.Y. De Winter, J.G. De Man, A. Loukas, M.S. Pearson, J.V. Weinstock, et al. 2009. Therapeutic potential of helminth soluble proteins in TNBS-induced colitis in mice. *Inflammatory Bowel Disease [Internet]* 15: 491–500. 10.1002/ibd.20787. Cited 23 Dec 2023. 10.1002/ibd.20787 19023900

[27] Rand, T.G., M. Sun, A. Gilyan, J. Downey, and J.D. Miller. 2010. Dectin-1 and inflammation-associated gene transcription and expression in mouse lungs by a toxic (1,3)-β-D glucan. *Archives of Toxicology [Internet]* 84: 205–20. 10.1007/s00204-009-0481-4. Cites 23 Dec 2023. 19904525 10.1007/s00204-009-0481-4

[28] Garside, P. 1999. Cytokines in experimental colitis. *Clinical and Experimental Immunology [Internet]* 118: 337. 10.1046/j.1365-2249.1999.01088.x. Cited 23 Dec 2023. 10594548 10.1046/j.1365-2249.1999.01088.x PMC1905439

[29] Coskun, M., S. Vermeire, and O.H. Nielsen. 2017. Novel targeted therapies for inflammatory bowel disease. *Trends in Pharmacological Sciences [Internet]* 38: 127–42. http://www.cell.com/article/S0165614716301584/fulltext. Cited 23 Dec 2023. 10.1016/j.tips.2016.10.014 27916280

[30] Chang, T.N., J.S. Deng, Y.C. Chang, C.Y. Lee, L. Jung-Chun, M.M. Lee, et al. 2012. Ameliorative effects of scopoletinfrom Crossostephium chinensis against inflammation pain and its mechanisms in mice. *Evidence-Based Complementary andAlternative Medicine* 2012: 595603. 10.1155/2012/595603. Cited 23 Dec 2023. 10.1155/2012/595603 PMC3443580 22991572

[31] Desreumaux, P., L. Dubuquoy, S. Nutten, M. Peuchmaur, W. Englaro, K. Schoonjans, et al. 2001. Attenuation of Colon Inflammation through Activators of the Retinoid X Receptor (Rxr)/Peroxisome Proliferator-Activated Receptor γ (Pparγ) HeterodimerA Basis for New Therapeutic Strategies. *Journal of Experimental Medicine [Internet]* 193: 827–38. http://www.jem.org/cgi/content/full/193/7/827. Cited 23 Dec 2023. 10.1084/jem.193.7.827 PMC2193371 11283155

[32] Nakajima, A., K. Wada, H. Miki, N. Kubota, N. Nakajima, Y. Terauchi, et al. 2001. Endogenous PPARγ mediates anti-inflammatory activity in murine ischemia-reperfusion injury. *Gastroenterology* 120: 460–469. 11159886 10.1053/gast.2001.21191

[33] Shah, Y.M., K. Morimura, and F.J. Gonzalez. 2007. Expression of peroxisome proliferator-activated receptor-γ in macrophage suppresses experimentally induced colitis. *American Journal of Physiology-Gastrointestinal and Liver Physiology [Internet]* 292: 657–66. 10.1152/ajpgi.00381.2006. Cited 23 Dec 2023. 10.1152/ajpgi.00381.2006 PMC1796914 17095756

[34] Dinarello, C.A. 2009. Immunological and Inflammatory Functions of the Interleukin-1 Family. *Annual Review of Immunology [Internet]* 27: 519–50. 10.1146/annurev.immunol.021908.132612. Cited 23 Dec 2023. 19302047 10.1146/annurev.immunol.021908.132612

[35] Bauer, C., P. Duewell, C. Mayer, H.A. Lehr, K.A. Fitzgerald, M. Dauer, et al. 2010. Colitis induced in mice with dextran sulfate sodium (DSS) is mediated by the NLRP3 inflammasome. *Gut [Internet]* 59: 1192–9. https://gut.bmj.com/content/59/9/1192. Cited 23 Dec 2023. 10.1136/gut.2009.197822 20442201

[36] Bashashati, M., R.H. Habibi, A. Keshavarzian, M. Schmulson, and A.K. Sharkey. 2023. Intestinal microbiota: a regulator of intestinal inflammation and cardiac ischemia?. Current Drug Targets 3: 199-208. https://www.eurekaselect.com/article/64686. Cited 23 Dec 2023. 10.2174/1389450116666150120104012 25601328

[37] Gerova, V.A., S.G. Stoynov, S. Katsarov, D.A. Svinarov, D.S. Katsarov, A. Keshavarzian, et al. 2011. Increased intestinal permeability in inflammatory bowel diseases assessed by iohexol test. *World Journal of Gastroenterology [Internet]* 17: 2211. https://www.ncbi.nlm.nih.gov/pmc/articles/PMC3092873/. Cited 23 Dec 2023. 10.3748/wjg.v17.i17. PMC3092873 21633531

[38] Laukoetter, M.G., M. Bruewer, and A. Nusrat. 2006. Regulation of the intestinal epithelial barrier by the apical junctional complex. *Current Opinion in Gastroenterology [Internet]* 22: 85–9. https://journals.lww.com/co-gastroenterology/fulltext/2006/03000/regulation_of_the_intestinal_epithelial_barrier_by.2.aspx. Cited 23 Dec 2023. 10.1097/01.mog.0000203864.48255.4f 16462161

[39] Kucharzik, T., S.V. Walsh, J. Chen, C.A. Parkos, and A. Nusrat. 2001. Neutrophil transmigration in inflammatory bowel disease is associated with differential expression of epithelial intercellular junction proteins. *American Journal of Pathology* 159: 2001–2009. 11733350 10.1016/S0002-9440(10)63051-9 PMC1850599

