# Supplementary material for: Exploring Scopoletin's Therapeutic Efficacy in DSS-Induced Ulcerative Colitis: Insights into Inflammatory Pathways, Immune Modulation, and Microbial Dynamics
Source: Inflammation. 2024 Jun 26;48(2):575–89. doi: 10.1007/s10753-024-02048-9 (PMC12053357; doi:10.1007/s10753-024-02048-9)
Supplement: Supplementary file 1 — Supplementary file1 (DOCX 209 KB) [file 10753_2024_2048_MOESM1_ESM.docx]

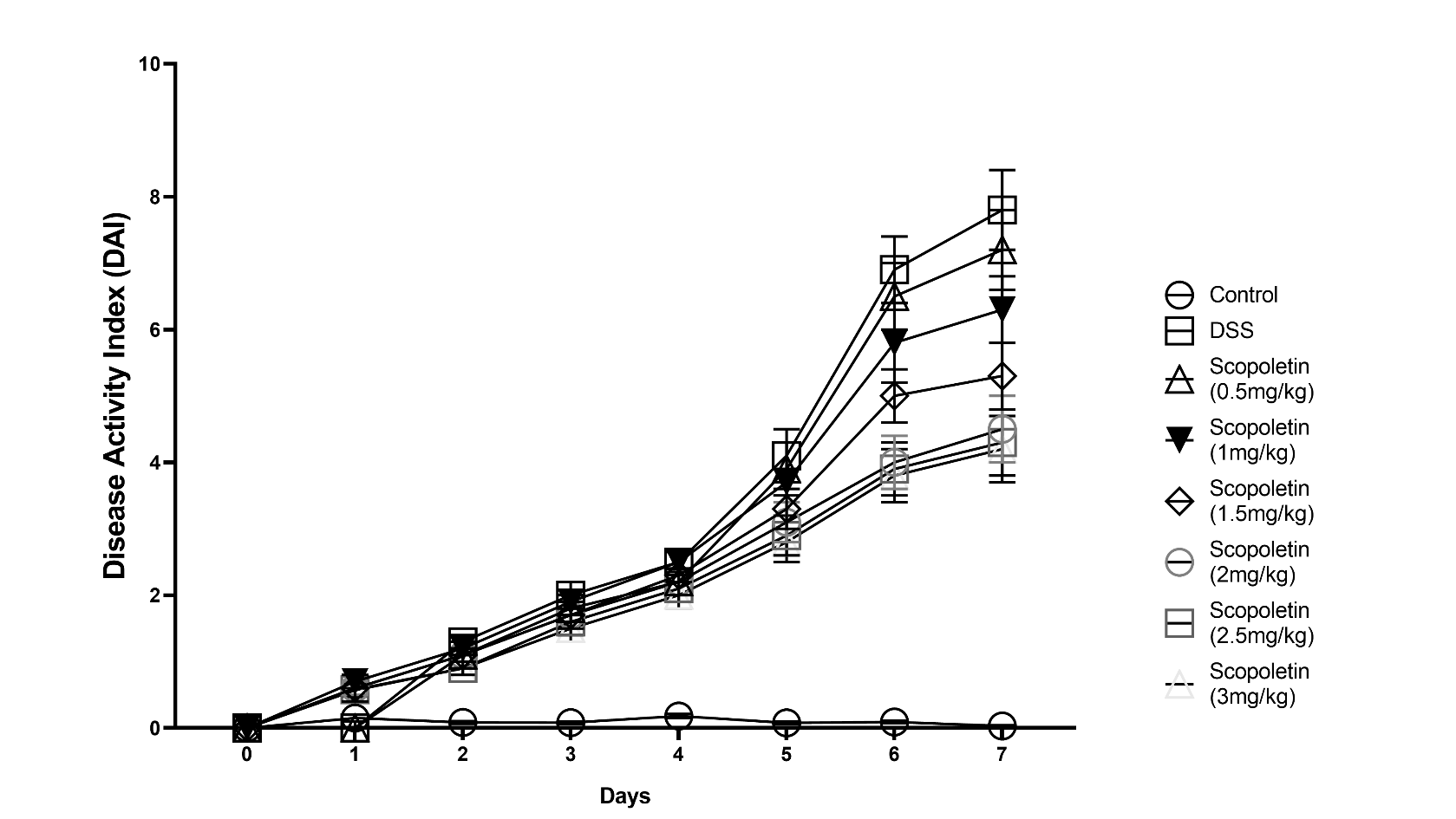


Supplumentary figure 1: The therapeutic effeicay of scopoletin was assessed using a wide range of doses. These preliminary investigations revealed that doses below 1 mg/kg were less effective in ameliorating colitis symptoms, whereas doses exceeding 2 mg/kg did not provide additional therapeutic benefits.
